# Inappropriate gestational weight gain impact on maternofetal outcomes in gestational diabetes

**DOI:** 10.1080/07853890.2022.2159063

**Published:** 2022-12-20

**Authors:** Sílvia Santos Monteiro, Tiago S. Santos, Liliana Fonseca, Miguel Saraiva, Fernando Pichel, Clara Pinto, Maria T. Pereira, Joana Vilaverde, Maria C. Almeida, Jorge Dores

**Affiliations:** a Division of Endocrinology, Diabetes and Metabolism, Centro Hospitalar Universitário do Porto, Porto, Portugal; b Division of Nutrition, Centro Hospitalar Universitário do Porto, Porto, Portugal; c Division of Obstetrics, Centro Hospitalar Universitário do Porto, Porto, Portugal; d On Behalf of the Pregnancy and Diabetes Study Group of the Portuguese Diabetes Society, Maternidade Bissaya Barreto, Coimbra, Portugal

**Keywords:** Gestational diabetes mellitus, gestational weight gain, large for gestational age, macrosomia, small for gestational age

## Abstract

**Objective:**

To evaluate the association between the dimension of deviation from appropriate gestational weight gain (GWG) and adverse maternofetal outcomes in women with gestational diabetes mellitus (GDM).

**Methods:**

We performed a multicentric retrospective study based on the Portuguese GDM Database. Women were classified as within GWG, insufficient (IGWG) or excessive (EGWG) than the Institute of Medicine recommendations. EGWG and IGWG were calculated for each prepregnancy BMI category. Large-for-gestational-age (LGA) and macrosomia were defined as a birthweight more than the 90th percentile for the gestational age and newborn weight greater than 4000 g, respectively. Logistic regression models (adjusted odds ratio [aOR] plus 95% confidence interval [95%CI]) were derived to evaluate the association between EGWG or IGWG and adverse maternofetal outcomes.

**Results:**

A total of 18961 pregnant women were included: 39.7% with IGWG and 27.8% with EGWG. An EGWG over 3 kg was associated with a higher risk of LGA infants (aOR 1.95, 95%CI 1.17–3.26) and macrosomia (aOR 2.01, 95%CI 1.23–3.27) in prepregnancy normal weight women. An EGWG greater than 4 kg was associated with a higher risk of LGA infants (aOR 1.67, 95%CI 1.23–2.23) and macrosomia (aOR 1.90, 95%CI 1.38–2.61) in obese women. In overweight women, an EGWG above 3.5 kg was associated with a higher risk of LGA infants (aOR 1.65, 95%CI 1.16–2.34), macrosomia (aOR 1.85, 95%CI 1.30–2.64), preeclampsia (aOR 2.40, 95%CI 1.45–3.98) and pregnancy-induced hypertension (aOR 2.21, 95%CI 1.52–3.21). An IGWG below −3.1 kg or −3kg was associated with a higher risk of small-for-gestational-age [SGA] infants in women with normal (OR 1.40, 95%CI 1.03–1.90) and underweight (OR 2.29, 95%CI 1.09–4.80), respectively.

**Conclusions:**

Inappropriate gestational weight gain seems to be associated with an increased risk for adverse maternofetal outcomes, regardless of prepregnancy BMI. Beyond glycemic control, weight management in women with GDM must be a focus of special attention to prevent adverse pregnancy outcomes.

**KEY MESSAGES**
- The dimension of deviation from appropriate gestational weight gain was associated with an increased risk for adverse maternofetal outcomes among women with gestational diabetes.
- Weight management must be a focus of special attention in women with gestational diabetes to prevent adverse pregnancy outcomes.

## Introduction

The association between maternal hyperglycemia and adverse maternofetal outcomes has long been known, constituting the primary focus on the management of women with gestational diabetes mellitus (GDM) in clinical practice [1–3]. However, the role of gestational weight gain (GWG) should not be underrated. GWG occurs mainly due to an increase in maternal body fat, water retention throughout gestation, maternal growth and development of the fetal-placental unit. Maternal weight gain varies by pregnancy trimester, the first half of pregnancy is characterized by a disproportionately high-fat deposition and the second half by progressive insulin resistance, exacerbated by maternal fat accumulation [4,5]. GWG has been identified as a strong and potentially controllable predictor of pregnancy and long-term health outcomes for both women and infants associated with several adverse maternofetal outcomes including preeclampsia, infant birth weight deviations (small for gestational age [SGA], large for gestational age [LGA] or macrosomia), preterm delivery and the need for cesarean section [6–8].

The Institute of Medicine (IOM) published recommendations on adequate gestational weight gain, specific for each prepregnancy body mass index (BMI) category [9]. These guidelines were based on the amount of weight gain during pregnancy necessary to achieve an ideal birth weight. A few studies have evaluated the link between GWG among BMI categories and adverse pregnancy outcomes, with inconsistent results reported [6–12]. A recent study by Mustaniemi et al. found that appropriate GWG protects against LGA and lowers birth weight standard deviation scores among women with GDM and prepregnancy obesity [11]. Given that IOM guidelines were only validated for healthy pregnant women, specific recommendations for GDM are necessary [12]. Given the potential synergistic effect between GWG and insulin resistance in this specific population, information regarding the optimal gestational weight gain is required [13].

Therefore, the aim of our study was to evaluate the real impact of the dimension of the deviation in GWG outside of IOM thresholds and maternofetal outcomes in women with GDM, as categorized by prepregnancy BMI.

## Methods

### Database and participants

This was an observational historical multicentric cohort study. We analyzed data from the Portuguese National Gestational Diabetes database, which includes data collected between the years 2011 to 2018 from 32 different hospitals located all around the country. Pregnant women were included in the study if they met any of the diagnostic criteria for GDM. Diagnosis and classification of GDM were performed according to the International Association of Pregnancy and Diabetes Study Group recommendations from 2010: fasting plasma glucose in the first trimester ≥ 92 mg/dl (5.1 mmol/l) but < 126 mg/dl (7.0 mmol/l) or 75-g oral glucose tolerance test between 24–28 weeks’ gestation ≥ 92 mg/dl (5.1 mmol/l) and/or ≥ 180 mg/dl (10.0 mmol/l) and/or ≥ 153 mg/dl (8.5 mmol/l), at fasting plasma glucose, 1 h and 2 h plasma glucose, respectively [14]. Pregnant women with twin pregnancy, pre-existing diabetes or overt hyperglycemia (fasting glucose ≥ 126 mg/dl or 2 h 75-g oral glucose tolerance test ≥ 200 mg/dl) at any time of gestation were excluded from the study.

### Study design and outcome

Gestational age was estimated from the last menstrual period and was either confirmed or corrected by ultrasonography. Prepregnancy BMI was calculated from self-reported prepregnancy weight and height. BMI categories were classified as follows: BMI <18.5 kg/m^2^ was classified as underweight, 18.5–24.9 kg/m^2^ as normal weight, 25.0–29.9 kg/m^2^ as overweight and ≥30.0 kg/m^2^ as obesity. Pregnant women were required to meet with their diabetes team regularly and weight was measured at every appointment. Total GWG (kg) was measured by calculating the difference between the last visit before delivery weight and prepregnancy weight. GWG before the 1st GDM appointment and between the 1st GDM appointment to delivery were also calculated. Women were classified as within, less or greater than IOM GWG recommendations for prepregnancy BMI category. IOM guidelines recommend an adequate GWG of 12.5–18.0 kg for underweight women, 11.5–16.0 kg for normal weight women, 7.0–11.5 kg for overweight women and 5.0–9.0 kg for obese women [9]. All pregnant women were evaluated fortnightly until 35 weeks of gestation and then weekly until delivery. Pregnant women with missing data on their prepregnancy weight and height or pre-delivery weight were excluded from analysis.

Pregnancy-induced hypertension was diagnosed as persistent blood pressure above 140/90 mmHg after 20 weeks of gestation among previously normotensive women and preeclampsia if accompanied by proteinuria ≥30 mg/mmol of creatinine [15]. Neonates were classified as LGA if their birth weight was ≥90th percentile for the gestational age, or SGA if their birth weight was <10th percentile for the gestational age, according to a Portuguese-validated birthweight standard [16]. Macrosomia was defined as a newborn weight greater than 4000 g. Prematurity was considered if the delivery occurred before 37 weeks of gestation. Congenital malformations were defined as any anatomical abnormality with either functional or esthetically relevant repercussions.

Excessive (EGWG) or insufficient gestational weight gain (IGWG) were defined as GWG greater or below the upper or lower range of IOM 2009 thresholds for each prepregnancy BMI category, respectively. These patients with EGWG or IGWG were then stratified according to the deviation from adequate GWG for each BMI prepregnancy category. Results were then compared to the development of adverse maternofetal outcomes: miscarriage, need for pharmacological treatment, pregnancy-induced hypertension, preeclampsia, polyhydramnios, neonatal hypoglycemia, neonatal hyperbilirubinemia, neonatal respiratory distress syndrome (RDS), admission to neonatal intensive care unit (NICU), SGA, LGA, macrosomia, premature delivery and congenital malformations.

### Statistical analysis

For continuous variables, distribution normality was tested through histogram observation and the Kolmogorov-Smirnov test. If normality was proven, results were presented as mean value ± standard deviation, if not they were presented as median value (25–75 percentiles). Qualitative variables were expressed as percentages and ratios. For each BMI prepregnancy category, patients were stratified over median EGWG or IGWG, with the subgroup closer to IOM recommendation targets designated as the reference group. The adjusted odds ratio (aOR) and 95% confidence interval (95% CI) used to evaluate the association between maternofetal outcomes and excessive or insufficient GWG were derived using multivariate logistic regression models. All analyses were further evaluated by stratifying potential confounder factors which were selected based on previous literature and biological plausibility such as maternal age, parity, previous GDM and length of gestation. Statistical analysis was performed using the IBM SPSS^®^ computer statistics program, version 25. Statistical significance was set at 95% level (*p* < 0.05).

### Ethical approval

This study is in accordance with the *Declaration of Helsinki* on medical protocol and ethics. Each participating hospital’s institutional review board approved data collection. Given the retrospective nature of this study and its anonymity, written consent was not required.

## Results

### Participants characteristics

Our study included a total of 18,961 pregnant women diagnosed with GDM. Over one-third with insufficient GWG (*n* = 39.7%; *n* = 7531) and about one-quarter (27.8%; *n* = 5275) with excessive GWG. IGWG and EGWG subgroups (*n* = 12,806) were then selected for the analysis and stratified according to each prepregnancy BMI category. Their baseline maternal and perinatal characteristics are summarized in Table 1. The distribution of the weight gain range among pregnant women with EGWG or IGWG is summarized in Table 2, stratified for prepregnancy BMI category. Median EGWG was 2.5 kg, 3 kg, 3.5 kg and 4 kg above the upper limit of IOM thresholds in the categories of underweight, normal weight, overweight and obesity, respectively. At 1st GDM appointment, a significant percentage of women with EGWG already exceeded the IOM criteria in each BMI category (17.5%, 14.1%, 22.0% and 23.5% in underweight, normal weight, overweight and obesity categories, respectively). On the other hand, the median IGWG was −3 kg, −3.1 kg, −3 kg and −3.5 kg below the lower limit in the categories of underweight, normal weight, overweight and obesity, respectively (Table 1).

**Table 1.** Background characteristics of the study population, stratified for BMI prepregnancy category.

Maternal and perinatal characteristics		Underweight	Normal weight	Overweight	Obesity
		(BMI < 18.5)	(BMI 18.5–24.9)	(BMI 25.0–29.9)	(BMI ≥ 30.0)
		*n* = 209	*n* = 5265	*n* = 3752	*n* = 3580
Maternal age, years		31 (27–36)	33 (30–37)	34 (30–37)	33 (30–37)
Primipara, *n* (%)		121 (57.8)	2654 (50.4)	1554 (41.4)	1295 (36.1)
Previous GDM, *n* (%)		17 (8.1)	504 (9.6)	500 (13.3)	560 (15.6)
Prepregnancy weight, kg		47 (45–50)	59 (55–63)	71 (67–76)	90 (83–98)
Prepregnancy BMI, kg/m^2^		17.8 (17.3–18.2)	22.5 (21.1–23.8)	27.2 (26.6–28.5)	33.8 (31.6–37.1)
GWG, kg		10.2 (8.0–12.0)	9.4 (7.0–11.2)	12.0 (4.6–15.1)	9.4 (1.5–13.0)
GWG until 1st GDM appointment, kg		6.4 (2.0–10.0)	5.5 (2.0–9.0)	5.0 (1.3–10.9)	2.6 (0–8.0)
GWG from 1st GDM appointment to delivery, kg		4.0 (1.0–7.5)	4.0 (1.0–7.5)	3.3 (0.8–7.5)	2.1 (0–6.0)
Preeclampsia, *n* (%)		5 (2.4)	92 (1.7)	103 (2.7)	344 (9.6)
Polyhydramnios, *n* (%)		5 (2.4)	111 (2.1)	96 (2.6)	92 (2.6)
Pregnancy-induced hypertension, *n* (%)		5 (2.4)	149 (2.8)	192 (5.1)	308 (8.6)
Cesarean section rate, *n* (%)		44 (21.1)	1522 (28.9)	1230 (32.8)	1454 (40.1)
Miscarriage, *n* (%)		1 (0.5)	14 (0.3)	9 (0.2)	6 (0.2)
Need for pharmacological treatment	Insulin, *n* (%)	47 (22.4)	1403 (26.6)	1252 (33.3)	1546 (43.2)
	Metformin, *n* (%)	11 (5.3)	377 (7.2)	557 (14.8)	730 (20.4)
Gestational age at birth, weeks		39 (38–39)	39 (38–39)	39 (38–39)	39 (38–39)
SGA, *n* (%)		49 (23.4)	679 (12.8)	366 (9.8)	292 (8.2)
Preterm birth, *n* (%)		26 (12.4)	376 (7.1)	262 (7.8)	226 (6.3)
LGA, *n* (%)		5 (2.4)	336 (6.4)	434 (11.6)	641 (17.9)
Macrosomia, *n* (%)		1 (0.5)	112 (2.1)	168 (4.5)	254 (7.1)
Neonatal hypoglycemia, *n* (%)		5 (2.4)	204 (3.9)	138 (3.7)	145 (4.1)
Neonatal hyperbilirubinemia, *n* (%)		18 (8.6)	538 (10.2)	402 (10.7)	405 (11.3)
Neonatal RDS, *n* (%)		2 (0.1)	143 (2.7)	88 (2.3)	119 (3.3)
Admission in NICU, *n* (%)		12 (5.7)	317 (6.0)	223 (5.9)	242 (6.8)
Birth trauma, *n* (%)		0	63 (1.1)	61 (1.6)	65 (1.8)
Congenital malformations, *n* (%)		5 (2.4)	155 (2.9)	116 (3.1)	102 (2.8)

BMI: Body mass index (kg/m^2^); GDM: Gestational diabetes mellitus; GWG: Gestational weight gain; kg: Kilograms; LGA: Large for gestational age; NICU: Neonatal intensive care unit; RDS: Respiratory distress syndrome; SGA: Small for gestational age. Results are shown as median (interquartile range) or number (percentages).

**Table 2.** Inappropriate gestational weight gain per BMI prepregnancy category.

BMI prepregnancy category	Excessive weight gain, kg (*n* = 5275)	*n*	Insufficient weight gain, kg (*n* = 7531)	*n*
Underweight	**+**2.5 (1.0–6.6)	40	**−**3.0 (1.5–4.6)	169
Normal weight	**+**3.0 (1.2–5.0)	1268	**−**3.1 (1.5–5.3)	3997
Overweight	**+**3.5 (1.5–6.3)	2151	**−**3.0 (1.5–5.0)	1601
Obesity	**+**4.0 (2.0–7.0)	1816	**−**3.5 (2.0–6.0)	1764

BMI: Body mass index; GDM: Gestational diabetes mellitus; kg: Kilograms. *Note*: *n* represents the total number of valid cases for each prepregnancy BMI category. Results are shown as median (interquartile range).

### Excessive gestational weight gain and adverse maternofetal outcomes

In normal weight women, an EGWG over 3 kg was associated with a higher risk of LGA infants (aOR1.95, 95%CI 1.17–3.26) and macrosomia (aOR 2.01, 95% CI 1.23–3.27). In the overweight category, an EGWG over 3.5 kg was also associated with a higher risk of LGA infants (aOR 1.65, 95% CI 1.16–2.64) and macrosomia (aOR 1.85, 95% CI 1.30–2.57) as well as a higher risk of preeclampsia (aOR 2.40, 95% CI 1.45–3.98), pregnancy-induced hypertension (aOR 2.21, 95% CI 1.52–3.21) and congenital malformations (aOR 1.91, 95% CI 1.05–3.48). In women classified as obese, an EGWG over 4 kg was associated with a higher risk of LGA infants (aOR 1.67, 95% CI 1.23–2.25) and macrosomia (aOR 1.90, 95% CI 1.38–2.61). We did not find any significant differences between EGWG and polyhydramnios, miscarriage, need for pharmacology treatment, cesarean delivery rate, neonatal hypoglycemia, neonatal hyperbilirubinemia, neonatal RDS, admission to NICU, or birth trauma. Complete data regarding maternal or fetal outcomes are depicted in Tables 3 and 4, respectively.

**Table 3.** Excessive gestational weight gain (EGWG) and adverse maternal outcomes.

		Underweight	Normal Weight	Overweight	Obesity
Maternal outcomes^a^		EGWG >2.5 kg	EGWG >3.0 kg	EGWG >3.5 kg	EGWG >4.0 kg
		aOR (95% CI)	aOR (95% CI)	aOR (95% CI)	aOR (95% CI)
Preeclampsia^b^		NA	1.71 (0.86–3.41)	**2.40 (1.45–3.98)**	1.29 (0.85–1.96)
Polyhydramnios^b^		NA	1.10 (0.56–2.15)	1.17 (0.71–1.92)	1.61 (0.92–2.83)
Pregnancy-induced hypertension^b^		NA	1.68 (0.93–3.02)	**2.21 (1.52–3.21)**	**1.37 (1.01–1.89)**
Cesarean section rate^c^		0.75 (0.19–3.11)	1.14 (0.90–1.46)	1.16 (0.97–1.40)	1.10 (0.91–1.34)
Miscarriage^d^		4.00 (0.82–19.59)	1.10 (0.84–1.43)	1.20 (0.99–1.45)	1.05 (0.86–1.29)
Need for pharmacological Treatment	Insulin	1.02 (0.21–5.03)	0.85 (0.65–1.11)	0.96 (0.80–1.15)	1.03 (0.85–1.24)
	Metformin	NA	1.05 (0.72–1.54)	1.18 (0.92–1.51)	1.08 (0.84–1.38)

EGWG: Excessive gestational weight gain; kg: Kilograms; NA: Not applicable. ^a^All analyses were based on a multivariate logistic regression model adjusted for maternal age, length of gestation, previous GDM and parity. Results are shown as adjusted odds ratio (aOR) plus 95% confidence interval (95% CI). ^b^Model also adjusted for chronic hypertension and macrosomia. ^c^Model also adjusted for macrosomia. ^d^Model also adjusted for previous miscarriage. In bold are presented the results significantly different from the estimate for the reference category (p < 0.05).

**Table 4.** Excessive gestational weight gain (EGWG) and adverse fetal outcomes.

	Underweight	Normal weight	Overweight	Obesity
Fetal outcomes^a^	EGWG > 2.5 kg	EGWG > 3.0 kg	EGWG > 3.5 kg	EGWG > 4.0 kg
	aOR (95% CI)	aOR (95% CI)	aOR (95% CI)	aOR (95% CI)
LGA^b^	NA	**1.95 (1.17–3.26)**	**1.65 (1.16–2.34)**	**1.67 (1.23–2.25)**
Macrosomia^b^	NA	**2.01 (1.23–3.27)**	**1.85 (1.30–2.64)**	**1.90 (1.38–2.61)**
Neonatal hypoglycemia	NA	0.91 (0.53-1.57)	1.30 (0.84-2.03)	0.98 (0.60-1.61)
Neonatal hyperbilirubinemia	1.06 (0.13–8.38)	1.09 (0.78–1.05)	1.04 (0.79–1.35)	1.10 (0.83–1.46)
Neonatal RDS	NA	0.91 (0.47–1.73)	0.89 (0.51–1.54)	1.44 (0.88–2.35)
Admission to NICU^c^	NA	1.15 (0.74–1.78)	0.95 (0.66–1.37)	1.32 (0.92–1.90)
Birth trauma^d^	NA	1.74 (0.76–4.01)	0.88 (0.49–1.89)	1.09 (0.58–2.03)
Congenital malformations^e^	NA	0.86 (0.47–1.58)	**1.91 (1.05–3.48)**	0.96 (0.56–1.63)

EGWG: Excessive gestational weight gain; LGA: Large for gestational age; NA: Not applicable; NICU: Neonatal intensive care unit; RDS: Respiratory distress syndrome. ^a^All analyses were based on a multivariate logistic regression model adjusted for maternal age, length of gestation, previous GDM and parity. Results are shown as adjusted odds ratio (aOR) plus 95% confidence interval (95% CI). ^b^Model also adjusted for previous macrosomia. ^c^Model also adjusted for birth weight, preterm birth, birth trauma, neonatal hypoglycemia, hyperbilirubinemia and RDS. ^d^Model also adjusted for previous macrosomia. ^e^Model also adjusted for preterm birth, previous history of miscarriage, previous history of congenital malformations and previous history of fetal or neonatal death. In bold are presented the results significantly different from the estimate for the reference category (p < 0.05).

### Insufficient gestational weight gain and adverse maternofetal outcomes

In normal weight women, an IGWG deviation over −3.1 kg was associated with a higher risk for preterm birth (aOR 1.55, 95% CI 1.23–1.96) and SGA infants (aOR 1.40, 95% CI 1.03–1.90). In the underweight category, an IGWG deviation over −3.0 kg was associated with a higher risk of SGA infants (aOR 2.02, 95% CI 1.04–4.67). In the overweight category, an IGWG deviation over −3.0 kg was associated with a lower risk for cesarean section (aOR 0.78, 95% CI 0.63–0.98). There was no association between IGWG and adverse maternofetal outcomes among pregnant women classified as overweight or obese. Complete data regarding maternal or fetal outcomes are depicted in Tables 5 and 6, respectively.

**Table 5.** Insufficient gestational weight gain (IGWG) and adverse maternal outcomes.

		Underweight	Normal weight	Overweight	Obesity
Maternal Outcomes^a^		IGWG <-3.0 kg	IGWG <-3.1 kg	IGWG <-3.0 kg	IGWG <-3.5 kg
		OR (95% CI)	OR (95% CI)	OR (95% CI)	OR (95% CI)
Preeclampsia^b^		0.93 (0.57–15.1)	1.09 (0.62–1.92)	1.41 (0.69–2.81)	1.39 (0.78–2.45)
Polyhydramnios^b^		0.52 (0.05–5.91)	0.95 (0.60–1.50)	1.00 (0.48–2.07)	1.11 (0.59–2.08)
Pregnancy-induced hypertension^b^		NA	1.19 (0.80–1.77)	0.80 (0.48–1.32)	0.74 (0.51–1.48)
Cesarean section rate^c^		0.86 (0.40–1.89)	0.93 (0.81–1.08)	**0.78 (0.63–0.98)**	0.84 (0.69–1.04)
Miscarriage^d^		1.33 (0.64–2.76)	1.12 (0.97–1.28)	0.98 (0.79–1.22)	0.91 (0.74–1.12)
Need for pharmacological Treatment	Insulin	1.76 (0.84–3.69)	1.12 (0.97–1.28)	1.10 (0.89–1.35)	1.12 (0.93–1.36)
	Metformin	1.65 (0.35–7.68)	1.03 (0.80–1.34)	0.93 (0.70–1.24)	1.20 (0.94–1.52)

IGWG: Insufficient gestational weight gain; kg: Kilograms; NA: Not applicable. ^a^All analyses were based on a multivariate logistic regression model adjusted for maternal age, length of gestation, previous GDM and parity. Results are shown as adjusted odds ratio (aOR) plus 95% confidence interval (95% CI). ^b^Model also adjusted for chronic hypertension and macrosomia. ^c^Model also adjusted for macrosomia. ^d^Model also adjusted for previous miscarriage. In bold are presented the results significantly different from the estimate for the reference category (p < 0.05).

**Table 6.** Insufficient gestational weight gain (IGWG) and adverse fetal outcomes.

	Underweight	Normal weight	Overweight	Obesity
Fetal outcomes^a^	IGWG <-3.0 kg aOR (95% CI)	IGWG <-3.1 kg OR (95% CI)	IGWG <-3.0 kg OR (95% CI)	IGWG <-3.5 kg OR (95% CI)
SGA^b^	**2.02 (1.04–4.67)**	**1.16 (1.04–1.38)**	1.02 (0.77–1.75)	1.16 (0.87–1.55)
Preterm birth^c^	1.07 (0.46–2.54)	**1.55 (1.23–1.96)**	0.88 (0.62–1.24)	1.14 (0.77–1.67)
Neonatal hypoglycemia	0.35 (0.04–3.46)	1.12 (0.80–1.54)	1.14 (0.67–1.95)	1.31 (0.83–2.07)
Neonatal hyperbilirubinemia	1.09 (0.36–3.27)	0.91 (0.74–1.12)	1.01 (0.72–1.41)	0.89 (0.65–1.21)
Neonatal RDS	1.08 (0.67–17.62)	1.27 (0.86–1.89)	1.49 (0.76–2.95)	1.11 (0.64–1.92)
Admission to NICU^d^	4.02 (0.59–27.31**)**	1.26 (0.96–1.65)	1.03 (0.68–1.56)	1.23 (0.83–1.81)
Birth trauma^e^	NA	1.71 (0.90–3.26)	1.33 (0.46–3.80)	0.83 (0.37–1.86)
Congenital malformations^f^	3.58 (0.36–35.30)	1.12 (0.76–1.64)	1.25 (0.70–2.24)	1.73 (0.93–3.24)

IGWG: Insufficient gestational weight gain; NA: Not applicable; NICU: Neonatal intensive care unit; RDS: Respiratory distress syndrome; SGA: Small for gestational age. ^a^All analyses were based on a multivariate logistic regression model adjusted for maternal age, length of gestation, previous GDM and parity. Results are shown as adjusted odds ratio (aOR) plus 95% confidence interval (95% CI). ^b^Model also adjusted for chronic hypertension. ^c^Model also adjusted for history of preterm birth. ^d^Model also adjusted for birth weight, preterm birth, birth trauma, neonatal hypoglycemia, hyperbilirubinemia and RDS. ^e^Model also adjusted for previous macrosomia. ^f^Model also adjusted for preterm birth, previous history of miscarriage, previous history of congenital malformations and previous history of fetal or neonatal death. In bold are presented the results significantly different from the estimate for the reference category (p < 0.05).

## Discussion

With this large population-based cohort study, we intended to verify if there was an increased risk of adverse maternofetal outcomes with the increase in the deviation from adequate gestational weight in pregnant women with GDM. According to our results, the association between GWG and perinatal morbidity shows increased risk with GWG. Excessive GWG was associated with higher infant birth weights and a higher rate of delivering an LGA infant, despite the prepregnancy BMI category, which suggests that prepregnancy BMI is likely not an effect modifier in the association between EGWG and perinatal morbidity. Our results are consistent with a recent systematic review of 23 studies, suggesting a relationship between excess GWG and perinatal morbidity to be a continuum as opposed to stepwise thresholds [17]. Excessive weight gain leads to fat mass deposition in the mother which is likely to exacerbate insulin resistance as pregnancy progresses and may explain these results. The association between total excessive maternal weight gain and the risk of macrosomia or delivering an LGA infant is well known, although the impact of EGWG during GDM management is less well understood [18–21]. Even with controlled glycemia, excessive gestational weight gain in women with GDM may lead to increased fetal growth due to an associated increase in the amounts of circulating amino acids, free fatty acids and triglycerides in the mother, which is not as easy to monitor as glycemia [22–23]. The strongest impact of excessive GWG was observed in women with a normal prepregnancy BMI. Accordingly, the 2009 IOM report stated the association between increased gestational weight gain and birth weight to be stronger in lower prepregnancy BMI categories, possibly due to a higher absolute weight gain in these subgroups [9]. We may speculate that normal weight women with excessive GWG have a higher increase in the risk of macrosomia and LGA babies when compared to women in the overweight and obesity categories because those with a higher BMI already present metabolic derangements and are thereby less affected by excessive gestational weight gain. Lastly, our sample size was inadequate to assess the impact of GWG on the women classified as underweight. This population may have additional risk factors that were not evaluated that could put their pregnancies at risk.

Interestingly, we also found that women in the overweight category who gained over 3.5 kg above IOM recommendations had a 2-fold increased risk of preeclampsia, pregnancy-induced hypertension and congenital malformations, although we did not verify this association among other BMI categories. Several studies have already proposed that preeclampsia may result from both a generalized inflammatory state and specific maternal metabolic conditions [24–26]. Excessive GWG probably results in an increased inflammatory response from the mother, with a transient higher risk for metabolic syndrome, therefore increasing the risk for preeclampsia. Moreover, women with preeclampsia usually experience an acute weight gain prior to delivery given their increased fluid accumulation. More studies are needed to clarify both the association and potential pathophysiology between excessive GWG and pregnancy-induced hypertension. Data regarding congenital malformations must be interpreted carefully, given the small number of neonates with this condition included (*n* = 69 in the EGWG group). Our results should be further validated in larger cohorts of infants from mothers in the overweight category and EGWG.

On the other hand, we identified a higher frequency of pregnant women with IGWG (40%) compared to previous studies among women with GDM (19–32%) [12,27]. Given that almost 45% had either overweight/obesity, we may theorize that this can result from rigorous lifestyle modifications with a strict diet and GDM management. In addition, we found women in the overweight category who gained less than 3 kg below IOM thresholds to have decreased risk for cesarean section, without increased risk of any maternofetal outcome analyzed in our study, although we did not verify this association in pregnant women classified as obese category. Nohr et al. showed lower GWG to be associated with a lower risk of LGA, cesarean section and postpartum weight retention in both pregnant women in the overweight and obesity categories [21]. Specifically, in women with GDM, some researchers have already declared that restricting GWG may benefit pregnancy outcomes whereas other researchers came to different conclusions [27–29]. Several studies did not find an association between GWG and birth weight in women with GDM and underweight or normal weight while others suggest that women with GDM with GWG below the guidelines were more likely to have SGA neonates [28–30]. In our study, both pregnant women with normal and underweight and GWG significantly below IOM recommendations were more likely to have SGA neonates. Normal weight GDM women with insufficient GWG also presented a higher risk for preterm delivery. Regarding higher prepregnancy BMI categories, we did not find an increased risk for adverse maternofetal outcomes in pregnant women with insufficient GWG, namely SGA. These results are consonant with our clinical approach to GDM pregnant women in overweight and obesity BMI categories, among which our primary focus is to prevent an EGWG, without considering a mandatory ‘minimal GWG’ among this subgroup of pregnant women. Moreover, within our sample, severe weight gain restrictions seem to be associated with poorer maternofetal outcomes only in pregnant women with underweight and normal weight.

This study has several limitations which could influence our results. First, its retrospective design should be recognized. To diminish the risk for either confounding or missing data biases, for each sub-analysis that was made, pregnant women with missing data were excluded. Although we used logistic regression models and stratification analyses to reduce confounding effects, there still might be unobservable and unmeasured confounders that we failed to account for in our analysis. Moreover, there might have been some changes in obstetric practice during our 8-year data collection period, at least in some of the institutions included. Another limitation to consider is the fact that prepregnancy BMI was calculated based on self-reported height and weight during the first antenatal visit. This may introduce recall bias, although it is widely used and validated in clinical research and allows comparison with other studies [8,31,32]. Moreover, mothers were measured at the first antenatal visit, thereby reducing the impact of reporting bias. This study was performed in a Caucasian population from the Mediterranean area and therefore our results should not be generalized to other populations. Lastly, given its multicentric nature, there was heterogeneity of people responsible for data collection. A coordinator was nominated to assure data uniformity

As for the strengths of our work, we emphasize our cohort sample size which is amongst the largest ever published in the field of GDM. Therefore, this large cohort of women provided adequate statistical power to examine rare perinatal morbidity. Few studies have investigated the impact of GWG outside IOM recommendations within GDM management on maternofetal outcomes. Further, as far as we know, no other study has assessed the impact of the dimension of deviation from IOM recommendations as we have done here.

## Conclusion

In this large population-based study, we found an association between the dimension of deviation in GWG from IOM recommendations and maternofetal outcomes. An excessive GWG was associated with macrosomia and LGA neonates, regardless of prepregnancy BMI category, and with preeclampsia/pregnancy-induced hypertension in pregnant women in the overweight category. Avoiding excessive GWG could be helpful in the prevention of these pregnancy outcomes. Severe weight gain restrictions were also associated with SGA neonates and preterm birth in lower prepregnancy BMI categories. Weight management in pregnant women with GDM highlights, even more, the need to prevent adverse pregnancy outcomes.

## Data Availability

The data cannot be shared for ethical/privacy reasons. The data that support the findings of this study cannot be shared publicly as it results from a population registry from the Portuguese National Gestational Diabetes database. The data will be shared on reasonable request to the corresponding author.
